# Highly Sensitive Multiplex Detection of Molecular Biomarkers Using Hybridization Chain Reaction in an Encoded Particle Microfluidic Platform

**DOI:** 10.3390/bios13010100

**Published:** 2023-01-06

**Authors:** Iene Rutten, Devin Daems, Karen Leirs, Jeroen Lammertyn

**Affiliations:** Department of Biosystems, Biosensors Group, KU Leuven, 3001 Leuven, Belgium

**Keywords:** biosensing, microfluidics, hybridization chain reaction

## Abstract

In the continuous combat against diseases, there is the need for tools that enable an improved diagnostic efficiency towards higher information density combined with reduced time-to-result and cost. Here, a novel fully integrated microfluidic platform, the Evalution™, is evaluated as a potential solution to this need. Encoded microparticles combined with channel-based microfluidics allow a fast, sensitive and simultaneous detection of several disease-related biomarkers. Since the binary code is represented by physically present holes, 2^10^ different codes can be created that will not be altered by light or chemically induced degradation. Exploiting the unique features of this multiplex platform, hybridization chain reaction (HCR) is explored as a generic approach to reach the desired sensitivity. Compared to a non-amplified reference system, the sensitivity was drastically improved by a factor of 10^4^, down to low fM LOD values. Depending on the HCR duration, the assay can be tuned for sensitivity or total assay time, as desired. The huge potential of this strategy was further demonstrated by the successful detection of a multiplex panel of six different nucleic acid targets including viruses and bacteria. The ability to not only discriminate these two categories but, with the same effort, also virus strains (human adenovirus and human bocavirus), virus subtypes (human adenovirus type B and D) and antibiotic-resistant bacteria (*Streptococcus pneumonia*), exemplifies the specificity of the developed approach. The effective, yet highly simplified, isothermal and protein-enzyme-free signal amplification tool reaches an LOD ranging from as low as 33 ± 4 to 151 ± 12 fM for the different targets. Moreover, direct detection in a clinically relevant sample matrix was verified, resulting in a detection limit of 309 ± 80 fM, approximating the low fM levels detectable with the gold standard analysis method, PCR, without the drawbacks related to protein enzymes, thermal cycling and elaborate sample preparation steps. The reported strategy can be directly transferred as a generic approach for the sensitive and specific detection of various target molecules in multiplex. In combination with the high-throughput capacity and reduced reagent consumption, the Evalution™ demonstrates immense potential in the next generation of diagnostic tools towards more personalized medicine.

## 1. Introduction

In the context of biosensors for medical diagnostics, a number of trends can be observed. Firstly, during the development of novel biosensing concepts there is a continuous search for biosensors that are able to detect specific disease-related biomarkers with increased sensitivity and specificity. This way, low amounts of these biomarkers can be detected, which is crucial for the prevention and early stage detection of many diseases (e.g., cancer or Alzheimer’s disease). Another trend in the diagnostic field focuses on the ability to interrogate multiple disease-related biomarkers with a single test, known as multiplexing. The analysis of multiple biomarkers at the same time allows for more detailed and reliable disease diagnosis and thus an improved outcome for the patient. Moreover, simultaneously testing several diseases drastically improves the diagnostic efficiency with respect to sample consumption, cost and time [1,2]. In a broader context, these advancements in the biosensing field align with cornerstones for achieving health-related targets set by the World Health Organization.

**Figure 1:**
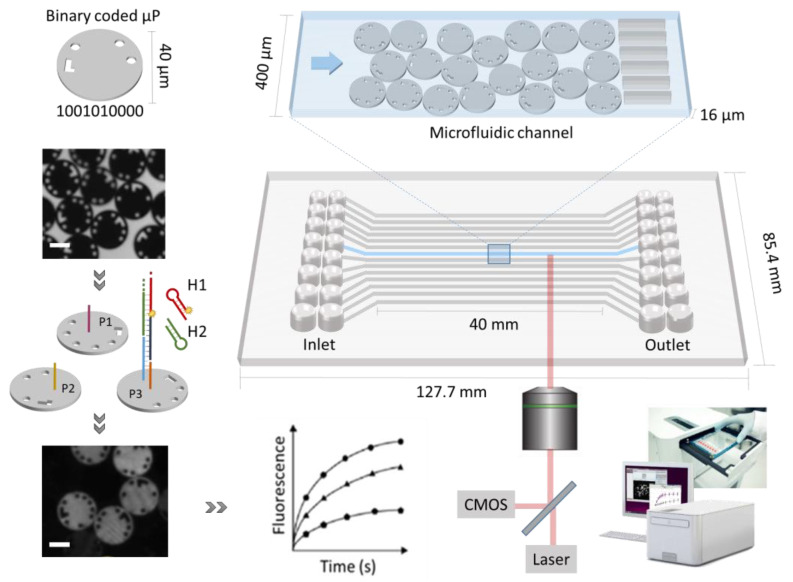
Schematic representation of the components of the Evalution™ technology, including digitally barcoded microparticles, microfluidic cartridge and an integrated instrument. Each microfluidic channel is embedded with encoded microparticles (P1, P2 and P3) that each serve as the substrate for the detection of a specific target. In presence of target, the hybridization chain reaction is initiated and the microparticles are identified during signal read-out. Based on bright field imaging, the microparticle populations are decoded and fluorescence imaging is used to quantify the signal. The scale bar represents 20 µm.

Two of the most commonly used approaches that enable high multiplex capacity for DNA or protein detection depend on x–y coordinates for coding of planar arrays (e.g., RayBiotech microarray) or color-coded microparticles (e.g., xMAP Luminex) [3]. Planar microarrays rely on a high spot density for the screening of high numbers of a target on a single slide. However, the flexibility of such an approach is often limited since the panel has to be defined prior to array manufacturing. Bead-based methods, on the other hand, are more flexible as the multiplex panel can be easily adjusted according to the assay application by coupling different capture molecules to different populations of beads. However, to be cost-efficient, these tests are often performed in batches (e.g., 96-well plates) requiring more sample [4]. Notwithstanding their commercial success, these techniques still demand considerable hands-on time. To reduce the handling time and move towards more automated platforms, microfluidics has been successfully combined in literature with these types of multiplexed detection [5,6,7]. In this context, an innovative and commercially oriented microfluidic Evalution™ platform was developed (MyCartis NV, Gent, Belgium) [4]. This platform creates a high-throughput multiplex environment by combining uniquely encoded microparticles with channel-based microfluidics (Figure 1) that allows for the integration of the entire assay workflow. This unique coding is represented by physical holes that go through the microparticle, which allows different codes to be created without spectral overlap or the risk of light-induced or chemical damage. In addition, the binary coded microparticles allow a high level of multiplex capacity, up to 1024 different codes and, together with the possibility to address 16 individual channels simultaneously, enable an elaborate collection of information [8,9]. The analysis costs related to a 150-plex analysis are estimated to be approximately EUR 10 per sample. Moreover, the short channel length, in combination with the narrow channel dimension (400 µm width and 16 µm height) results in a low sample consumption in the microliter range. Although the binary coding of the Evalution™ platform provides a higher encoding capacity compared to conventional methods that rely on spectral target discrimination [10], its ability to detect low concentrations of biomarker currently remains unexplored.

**Figure 2:**
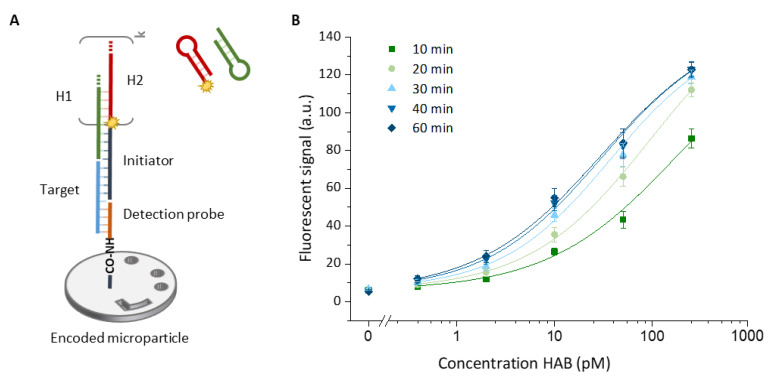
(**A**) Schematic representation of the assay build-up. First, the target (light blue) is captured by the detection probe (orange), which is immobilized on the surface of an encoded microparticle. After capturing the target, the polymerization of two metastable hairpin structures, called H1 (green) and H2 (red), is initiated by the initiator probe (dark blue). The length of the resulting nicked ds-DNA construct (i.e., the amount of signal amplification) depends on the number of HCR cycles (k) that increase over time. (**B**) Calibration curves obtained for the detection of the HAB target (250, 50, 10, 2, 0.4 pM and blank) by employing different durations of the HCR ranging from 10 to 60 min. Error bars indicate one standard deviation (*n* = 3).

In order to achieve the high sensitivity, necessary to detect low target concentrations, signal amplification strategies can be used. Two main strategies for signal amplification can be discerned: the signaling molecules (1) can diffuse or flow away from the signal amplification system or (2) remain tethered to it. The latter is of particular interest when the bioassay is directly exposed to the flow of liquid, as is the case for the Evalution™. To achieve this localized accumulation of signaling molecules, DNA-based nanostructures can be used. While formation of a DNA construct often is protein-enzyme mediated (e.g., rolling circle amplification [11,12]), isothermal amplification techniques can still suffer from the drawbacks related to the limited stability of the enzymes and their stringent buffer requirements. As an alternative, a protein-free DNA amplification strategy, hybridization chain reaction (HCR), was developed by Dirks and Pierce [13]. HCR is an enzyme-free, isothermal and robust amplification technique of low complexity and therefore easy to use. The key driving force of HCR is a pair of DNA hairpins that propagate a chain reaction of hybridization events. In a typical HCR, the target initiates a toehold-mediated strand displacement, triggering a cascade reaction that consists of the cross-opening of two meta-stable DNA hairpins (H1 and H2, in Figure 1 and Figure 2A). This reaction results in the assembly of a nicked double-helix construct [14,15]. Detection of the target can be achieved by using hairpin structures that are modified with signaling molecules [16]. This way HCR has been successfully employed as a signal amplification technique in several solid-phase biosensing applications (e.g., electrochemistry) for nucleic acid target detection [14,16,17]. Although these applications provide already evidence that by using HCR as a signal amplification technique for solid-phase assays, the detection limit can be improved by several orders of magnitude (100-fold on average); so far, this has never been demonstrated in a microfluidic system in combination with multiplexed solid-phase assays [14].

Benefiting from the advantages of both systems, in this paper we combine, for the first time, HCR as an amplification strategy with encoded microparticles in a microfluidic platform for the sensitive multiplex detection of six different molecular biomarkers, without the aforementioned drawbacks related to spectral encoding techniques, protein enzymes, thermal cycling or non-automated systems. To this purpose, a panel of six different nucleic acid targets, derived from the genetic material of both pathogenic bacteria and viruses causing respiratory tract infections, is used as a model system. The selected target sequences demonstrate the ability to specifically recognize and discriminate: viruses from bacteria, virus subtypes (human adenovirus type B and D) [18], virus strains (human adenovirus and human bocavirus) [19] and antibiotic-susceptible bacteria strains from resistant strains (*Streptococcus pneumonia*) [20]. To obtain a generic approach suitable for the multiplexed detection of a wide variety of target molecules, the HCR initiators are designed to match with a sole hairpin-ensemble. Moreover, the robustness of the system in clinically relevant matrices will be demonstrated in spiked nasopharyngeal swab samples. As such, we will evaluate the Evalution™ platform combined with HCR as a universal signal amplification tool to achieve highly sensitive and high-throughput multiplexed target detection and quantification.

## 2. Materials and Methods

### 2.1. Reagents

Reagents were of analytical grade and purchased from Sigma-Aldrich (Diegem, Belgium) unless stated otherwise. All unmodified, amino-modified and fluorescently labeled oligonucleotides were purchased from Integrated DNA Technologies (IDT, Haasrode, Belgium). Each of these sequences can be found in Supporting Information (Table S1).

### 2.2. Evalution™ Instrument

The Evalution™ (MyCartis, Gent, Belgium), a microfluidic platform, relies on encoded microparticles [4]. These disk-shaped silicon microparticles (40 µm diameter and 10 µm height) carried a 10-bit digital code that allowed identification. The microparticles were modified with carboxyl groups that allowed functionalization with bioreceptors. Different bioreceptors were immobilized on differently encoded microparticle populations (i.e., particles with identical coding belonged to the same population). A mixture of these pre-functionalized microparticles was then loaded into a microfluidic cartridge for multiplex detection. The cartridge has a size of 127.7 × 85.4 mm, similar to a standard 96-well plate. This cartridge, accommodating 16 individual microfluidic channels, was inserted into the instrument. The microfluidic flow was controlled by applying a pressure difference (set to 300 mbar) over the in- and outlet of each of the channels. This pressure difference yielded a flow rate of approximately 30 nL/s. More technical details can be found in Falconnet et al., 2015 [4]. In addition, the temperature in the channels was controlled, ranging from 25 °C to 55 °C. At the start of each experiment, the background fluorescence signal of the microparticles was determined by scanning each of the channels from the bottom of the cartridge as depicted in Figure 1. The imaging system that was integrated in the instrument relied on a 10× objective (NA 0.3) and a highly sensitive CMOS camera, mounted on an automated stage that moves in x, y and z directions to scan the channels. Each channel is scanned sequentially and in multiple fields of view (each containing approximately 240 microparticles). Each microparticle generated over 1800 results corresponding to each of the pixels. After the first scan, the required assay steps were performed, which include target incubation, washing and fluorescent signal generation. Visualization of the fluorophores (ATTO 550) was obtained through exposure to a green laser (532 nm, 20 mW and 1000 ms), also incorporated in the instrument. The recorded signals were linked to the corresponding particle codes that were precedingly determined in brightfield. In summary, a typical assay was performed in the following steps: (1) microparticle functionalization (Section 2.3), (2) loading of the microparticle mixture into the microfluidic cartridge (Section 2.4), (3) insertion of the microfluidic cartridge in the integrated instrument to perform a predefined protocol (Section 2.5, Section 2.6 and Section 2.7).

### 2.3. Microparticle Functionalization

Each batch of uniquely coded microparticles (µP) (MyCartis, Gent, Belgium) was resuspended in 200 µL of nuclease free water and washed three times in 500 µL of activation buffer (100 mM MES, 0.3% Tween 20, pH 5). Subsequently, the carboxyl groups on the microparticle surface were activated by adding a mixture of 500 µL of 10 mg/mL sulfo-NHS solution and 100 µL of 50 mg/mL EDC solution, both prepared in activation buffer. The homogenized microparticle suspension was incubated for 40 min at 1100 rpm and 22 °C. Following the activation, the microparticles were washed three times with coupling buffer (100 mM MES, 0.3% Tween 20, pH 5.8). Afterwards, the activated microparticles were incubated with 3 µM of amino-modified DNA probes (detection probes) in 600 µL coupling buffer for 40 min at 1100 rpm and 22 °C. The detection probe sequences can be found in Supporting Information (Table S1). In a final step, the microparticles, functionalized with detection probes, were washed three times in PBST buffer (137 mM NaCl, 2.7 mM KCl, 10 mM Na_2_HPO_4_, 1.8 mM KH2PO4, 0.3% Tween 20, pH 7.4), aliquoted and stored at −20 °C until further use.

### 2.4. Microfluidic Cartridge Loading

The functionalized microparticles were loaded into the channels of the microfluidic cartridge in a semi-automated fashion. To this purpose, a loading station (MyCartis, Gent, Belgium), optimally exploiting gravitational forces, was used. In combination with the filter structure and the end of the channels, the microparticles form a static monolayer in the detection zone as schematically represented in Figure 1. Moreover, the microparticles are equipped with supporting units on the top to allow sufficient flow between the channels surface and the microparticle surface. First, a microparticles mixture was prepared in PBST buffer with a concentration of 5000 µP/mL. This mixture comprised multiple uniquely coded microparticle populations, depending on the required multiplex factor of the test. Prior to loading, the channels and inlet wells were prewetted with ethanol (20 µL), followed by PBST buffer (110 µL). The channels were loaded using 100 µL of the microparticle mix per channel. Depending on the multiplex factor, the channels were filled with the microparticle mixture, ensuring that a minimum of 30 microparticles represented each microparticle population per channel. Finally, the solution in the inlet wells was replaced with 100 µL of storage buffer, after which the microfluidic cartridge was inserted into the Evalution™ instrument.

### 2.5. Singleplex Assay Characterization

The effect of the HCR amplification on the recorded signals was investigated based on the human adenovirus type B (HAB) target. Target detection was achieved through following steps: (1) 20 min incubation with different target concentrations (250, 50, 10, 2, 0.4 pM and blank), (2) 20 min incubation with 1 µM excess of HAB initiator and (3) 60 min incubation with a 1 µM mixture of hairpin 1 (H1) and fluorescently labeled hairpin 2 (H2). During this last incubation, the fluorescent signal was recorded with 5 min intervals. All incubation steps were performed in SPSC buffer (1 M NaCl, 50 mM Na2HPO4, 0.05% Tween 20, pH 7.5) at 25 °C. The assay steps were separated by a 2 min wash with SPSC buffer (0.3% Tween 20). In a similar way a reference signal (i.e., non-amplified signal) was established. Here, to ensure only one HCR cycle to occur, resulting in only one fluorescent label per target molecule, H1 and H2 were flowed sequentially for 20 min and separated by a washing step.

### 2.6. Multiplex Detection in Buffer

The detection of a panel of 6 different targets was performed in multiplex format. The investigated targets included human adenovirus type B (HAB), human adenovirus type D (HAD), human bocavirus (HB), *Streptococcus pneumonia* (SP) and two antibiotic-resistant strains of *Streptococcus pneumonia* (SPR1 and SPR2). First, the occurrence of cross-reactivity was studied and challenged by applying elevated temperatures during target incubation, ranging from 35 to 55 °C with 5 °C intervals for a target concentration of 250 pM. Next, multiplex target detection (250, 50, 10, 2, 0.4 pM and blank) was performed at 45 °C using the same assay configuration as described in the previous paragraph for the singleplex detection using HCR. To address all 6 targets at once, an initiator mixture of 1 µM, containing all initiator probes, was used.

### 2.7. Detection in Nasopharyngeal Swabs

Following the protocol described in the previous paragraphs, the detection of human adenovirus type B (HAB) was evaluated in nasopharyngeal samples, thereby mimicking a respiratory tract infection to be identified using the developed multiplex model case presented in this work. The used swab samples were obtained from healthy donors and eluted in 3 mL of universal transport medium (Copan Flock technologies S.R.L., Brescia, Italy). For further analysis, the samples were diluted 10-fold in SPSC buffer and subsequently spiked with HAB target at the same concentrations as in buffer. In addition, 0.5% (SDS) was added to the sample for lysis purposes. This procedure was based on the procedure previously described in Leirs et al., 2016 [21].

### 2.8. Data Analysis

The average fluorescence intensity for each microparticle population was recorded using the supplied software (Evalution Control 4.2, MyCartis, Gent, Belgium). Both end-point measurements as well as measurements over time were performed. In the latter situation, fluorescent label was present in the channels, causing a background signal. This constant channel background was subtracted from the average fluorescent signal obtained after HCR. Data were further processed using Origin 7 (OriginLab, Northampton, MA, USA). Calibration curves were fitted with a four-parameter logistic fit. LOD values were calculated based on the statistical method described by Holstein et al. [22]. The LOD is estimated using information of both blank and test samples. A more detailed description of the formulas that were used can be found in Supporting Information. This method generates a 95% confidence interval for the calculated LOD values that allows a statistical comparison of the obtained values [22].

## 3. Results

### 3.1. Singleplex Assay Characterization

To generate a signal upon target binding, three sequential assay steps are required: (1) capturing of the target by the detection probe tethered to the surface of an encoded microparticle, (2) binding of the initiator, providing the starting point for the HCR reaction and (3) HCR reaction by incubating with a mixture of metastable hairpin structures (H1 and H2), of which H2 carries a fluorescent label. The short loop region and long stem region of the hairpins ensure a thermodynamic steady state of the two hairpins in solution [15]. This steady state is only disturbed by the hybridization of the initiator (if present) to H1, resulting in an alternating addition of the two hairpins to the nicked double-helix construct. A schematic of the assay construct is depicted in Figure 2A.

In a typical HCR system, hairpin polymerization is initiated by the target sequence [16]. This requires a target-depending redesign of each of the hairpin sequences. To develop a universal detection system, capable of specifically labeling different targets, first the initiator probes were redesigned instead of the two hairpin sequences. These probes were equipped with a sequence part that allows specific hybridization to the target, while leaving the other end, initiating the hybridization chain reaction, unaltered and hence uniform for each target. The resulting sequences can be found in Supporting Information (Table S1).

**Figure 3:**
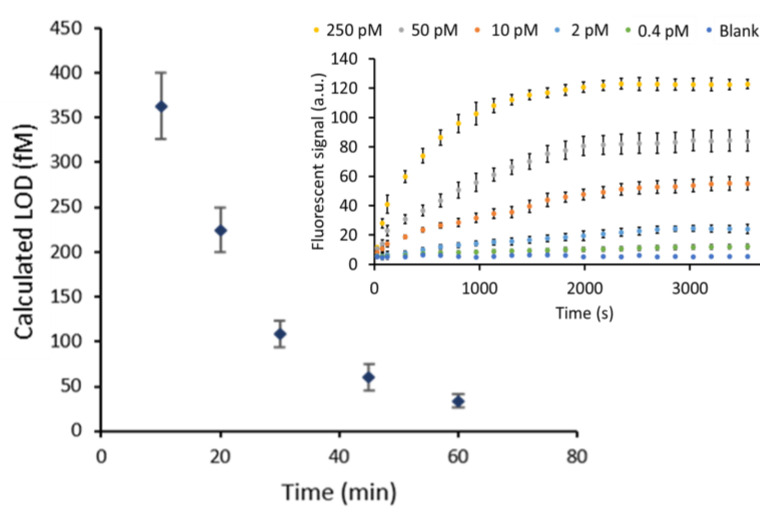
The calculated LOD values (fM) obtained for varying HCR polymerization times. The error bars represent the estimated LOD range. Insert: HCR reaction over time for different HAB target concentrations (250, 50, 10, 2, 0.4 pM and a blank). The error bars represent the standard deviation of three repetitions. The SNR ratios that were mentioned in the text are based on the highest concentration of HAB target that is indicated by the yellow labels. A high resolution version of the inset can be found in supporting Figure S1.

We first verified the performance of the HCR system in the microfluidic environment of the Evalution™ platform. We evaluated the performance for the singleplex detection of the HAB target for a 5-fold target dilution series between 250 pM and 0.4 pM (Figure 2B). To more thoroughly understand the effect of the HCR reaction kinetics on the obtained fluorescent signal, different polymerization times were tested ranging from 10 min up to 60 min, yielding to increasingly more and on average longer HCR products and resulting in different levels of signal amplification. The highest signals were obtained after 60 min of HCR, although the difference between 50 and 60 min was no longer significant; that is why, for a clear visual representation in Figure 2B, the results for 50 min are not shown. The full dataset corresponding to Figure 2B can be found in Supporting Information Table S2. Although the signal intensity saturates over time, the overall signal intensity reaches the highest level for longer amplification times (Figure 3 insert and Figure S1). In the absence of the target, no significant increase in signal was recorded over time, demonstrating the specificity of the amplification system (Figure 3 insert). This observation translated into an increase in the overall signal-to-noise ratio for longer amplification times. This ratio almost doubled when the amplification time was increased from 10 to 60 min, corresponding to a signal-to-noise ratio of 12 and 23, respectively, for the 250 pM concentration of the HAB target. The signals obtained for different polymerization durations were fitted with a four-parameter logistic model, which is well-known to be suitable for biological systems [22]. Fitting these models resulted in an adjusted fitting parameter (R^2^) of ≥0.975 for all cases. The difference in assay performance, already indicated by the increasing overall signal-to-noise ratio, was further confirmed when, based on these fittings, the LOD values were calculated (Figure 3).

Using the statistical method described by Holstein et al., the LOD together with its corresponding 95% confidence interval LOD was determined. After 10 min of amplification a calculated LOD of 362 ± 37 fM was reached. Performing the HCR reaction for 30 min resulted in an LOD of 108 ± 20 fM. It was observed that the rate at which the calculated detection limit improves decreases for longer amplification times. In spite of this decreased effect on the sensitivity (which refers to LOD in this paper), after 60 min the calculated LOD value reached 34 ± 11 fM, a 10-fold decrease compared to the value obtained after only 10 min of amplification.

To truly assess the added value of signal amplification by HCR, the obtained results were compared to a non-amplified reference system. This reference was established by flowing each of the two hairpin sequences sequentially instead of at the same time. This sequential flow, separated by a washing step, allowed full control of the number of HCR cycles, which in this case was limited to only a single cycle (k = 1). After one cycle of HCR, only one fluorescent label was incorporated, which corresponds to maximally one fluorescent label per HAB target. Fitting the resulting calibration curve (Figure S2) led to a calculated LOD of 890 ± 110 pM. This showed an impressive 2.4 × 10^3^-fold and 2.6 × 10^4^-fold improvement of the LOD after 10 min and 60 min of HCR time, respectively. The high level of amplification reached after only a limited reaction time can be attributed to the advantage of having a continuous flow, providing a constant supply of fresh reagents [4]. These findings demonstrate the potential of this HCR-based amplification method for highly sensitive target detection.

### 3.2. Multiplex Detection in Buffer

**Figure 4:**
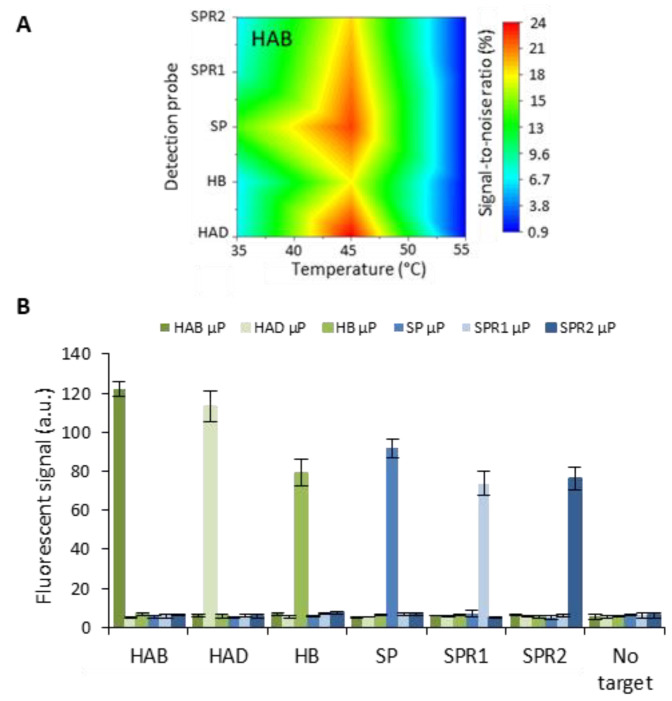
(**A**) Color map visualizing the signal-to-noise ratio for HAB target capturing at different temperatures. (**B**) Fluorescent signal for six different target sequences HAB, HAD, HB, SP, SPR1 and SPR2) obtained for a concentration of 250 pM target and a blank containing no target. Each channel, containing six different microparticle (µP) populations, was incubated with a sole target at 45 °C. The error bars represent one standard deviation (*n* = 2).

After achieving successful singleplex detection, the performance of the HCR system was evaluated in a multiplex context. A panel of six different target sequences was used as a model system, including human adenovirus type B and D, human bocavirus and wild-type *Streptococcus pneumonia*, together with two of its antibiotic-resistant strains. One very important aspect to consider when performing multiplex detection in a single channel is the occurrence of cross-reactivity. In the particular case of nucleic acid detection, temperature can be used as a tool to avoid unwanted interactions while specific interaction can still occur. An elevated temperature increases the stringency that drives strand hybridization to the most thermodynamically stable combinations. Equilibrium base-pairing simulations (predicted by the NUPACK analysis software, developed by Pierce et al. [23]) and preliminary experiments at a temperature of 25 °C indicated that the majority of non-specific signal was caused by the non-specific interaction of the target with the detection probes rather than unwanted interactions of the initiator or hairpins (Figure S3). Based on these insights, the signal-to-noise ratios were analyzed for different temperatures (ranging from 35 °C to 55 °C with 5 °C intervals) for target concentrations of 250 pM. Color maps, exemplified in Figure 4A for the HAB target and further included in Supporting Information (Figure S4) show a maximal signal-to-noise ratio when the temperature was elevated to 45 °C during the interaction of the target with the detection probes immobilized onto the microparticle (µP) surface. The corresponding fluorescent signal (Figure 4B) clearly showed a high specific interaction with a negligible amount of non-specific binding.

**Figure 5:**
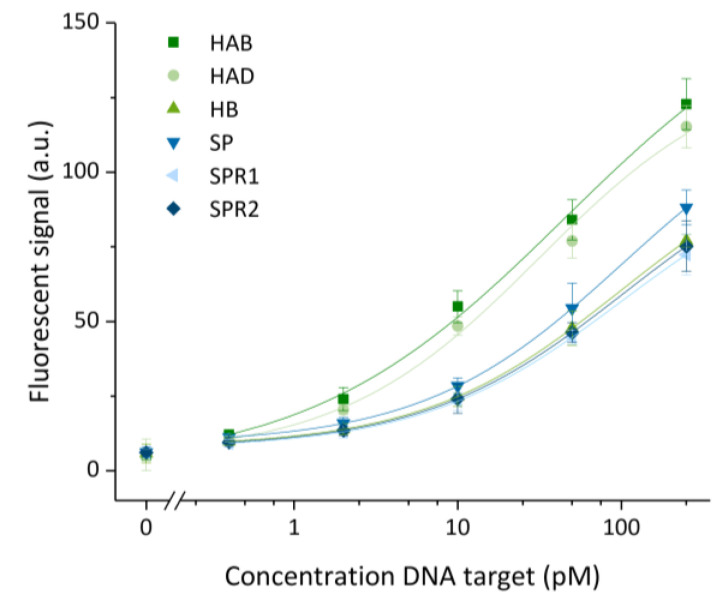
Calibration curves obtained for six different targets (HAB, HAD, HB, SP, SPRR1 and SPR2) for a 5-fold diluted concentration range from 250 to 0.4 pM, including a blank. Hybridization chain reaction was performed for 60 min. Data were fitted with an adjusted R^2^ of ≥0.98 for all targets. The error bars represent one standard deviation (*n* = 3).

After optimization of the assay towards a maximized signal-to-noise ratio, the aforementioned condition was used to perform further experiments. Six calibration curves were established, one for each target in a 5-fold dilution ranging from 250 pM to 0.4 pM and a blank (Figure 5). For this test, HCR was performed at 45 °C for the maximal duration of 60 min to reach the highest sensitivity possible within an acceptable timespan and assuming a similar behavior for each target. A difference in assay performance was noticed for different target sequences. This difference might be attributed to the variation in the target and detection sequences. The highest signal was obtained for the sequences with the highest GC content, being >55% for HAB and HAD. The SP, SPR1 and SPR2 targets had a similar GC content of 30–33%, and detection of these targets also resulted in a similar performance. The HB target seems to be an exception that in spite of a GC content of 46% gave rise to lower signals than targets with a lower GC content. This observation can be attributed to the strong secondary structure of the HB target (predicted by NUPACK) as this creates a higher energy barrier to intermolecular hybridization [24]. The influence of the GC content on the binding capability is furthermore exemplified by the difference in the calculated LOD values. The values obtained after fitting the four-parameter logistic model were 33 ± 4, 51 ± 5; 106 ± 9, 88 ± 6, 151 ± 12 and 136 ± 11 fM for HAB, HAD, HB, SP, SPR1 and SPR2, respectively. The obtained LODs varied in correspondence with the overall intensity of fluorescent signal: a higher specific signal resulted in a lower LOD. The relation between the sequence and base-pair hybridization efficiency indicates that similar or even better overall LOD values could be obtained by choosing different (e.g., having higher GC content or weak secondary structures) or longer sequences related to the genetic sequence of the target. Moreover, varying the amplification time offers the possibility to tune the assay for either assay speed, sensitivity or LOD, as desired.

### 3.3. Detection in Nasopharyngeal Swabs

**Figure 6:**
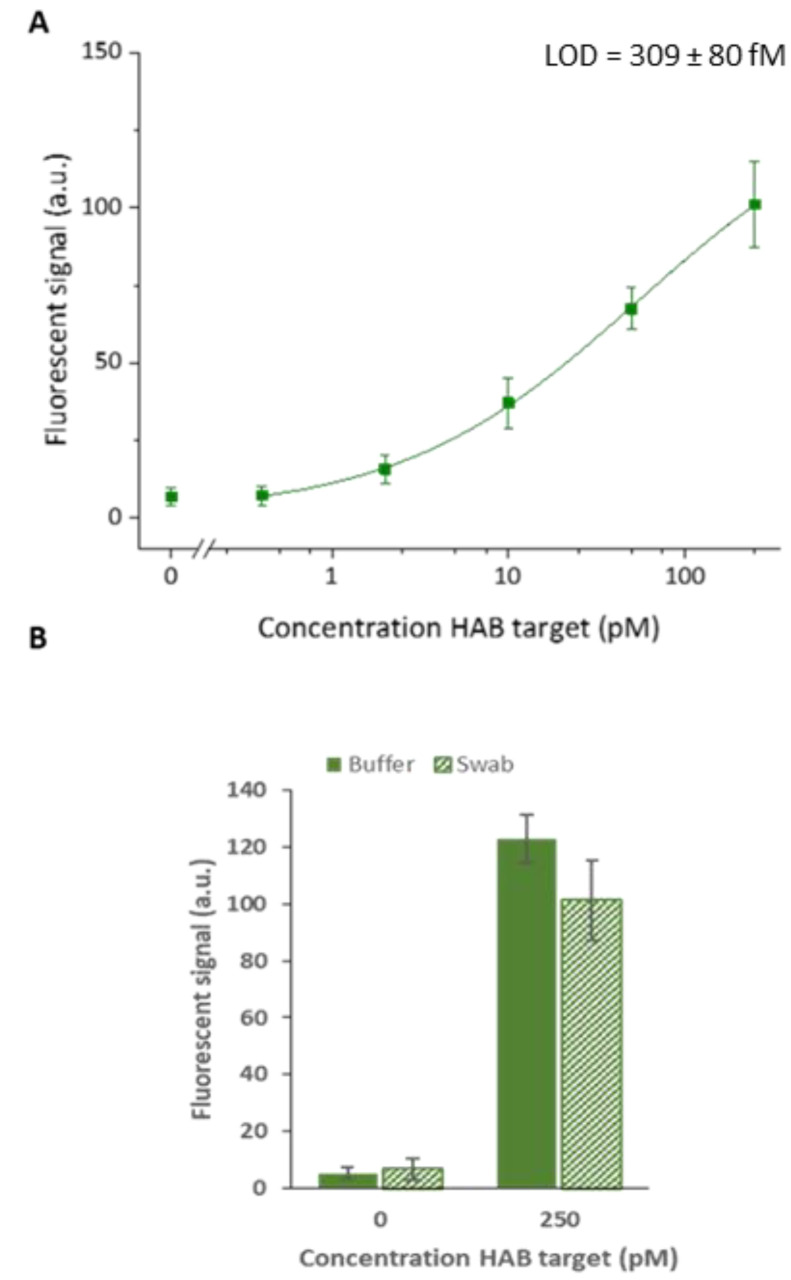
(**A**) Calibration curve obtained for HAB target detection in 10-fold diluted nasopharyngeal swab samples spiked with 250, 50, 10, 2, 0.4 pM and a blank. The calculated LOD is also indicated in the figure. (**B**) Comparison of the signal to buffer sample. Hybridization chain reaction was performed for 60 min. The error bars represent one standard deviation (*n* = 3).

Considering the importance of target detection in clinical samples, the performance of the developed system was evaluated in a relevant sample matrix. Since each of the studied targets are pathogens related to respiratory tract infections, detection is typically performed in nasopharyngeal swab samples. To this purpose, nasopharyngeal swabs, obtained from healthy donors, were diluted 10-fold in the SPSC buffer with 0.5% SDS for lysis [21]. This sample matrix was spiked with HAB target concentrations of 250, 50, 10, 2 and 0.4 pM, similar to the assay in the buffer. Detection of the target was carried out directly in this complex matrix. To obtain the highest sensitivity possible within an acceptable timespan and allow comparison to the results obtained in the buffer, HCR was performed for 60 min at 45 °C. Using the four-parameter logistic model, the resulting calibration curve was fitted with an adjusted R^2^ of 0.99 (Figure 6). Direct detection of the target in 10-fold diluted swab samples resulted in a lower overall fluorescent signal when compared to target detection in the buffer. This can be attributed to the influence of the complex sample matrix environment on the detection efficiency. More specifically, this could be explained by the molecules present in the sample matrix, which interact with the target, reducing the amount available for assay detection. Another explanation for the overall reduced signal intensities can be found in the suboptimal buffer conditions resulting in a loss in the overall hybridization efficiency between detection and target probes. Despite the loss in signal, a calculated detection limit of 309 ± 80 fM was obtained. However, the experimental data showed that 400 fM, which was the lowest concentration tested, could not be distinguished from the blank. Hence, the calculated LOD is slightly overestimated. In addition, it was observed that the 10-fold diluted swab sample did not cause a significant increase in non-specific interactions. This is an important aspect as enhanced non-specific interactions could induce unwanted false positive results. Moreover, the obtained result approximates the magnitude of the in-literature described detection limit of 16.6 fM for human adenovirus target detection through traditional PCR [18]. Considering the successful detection of the target in a complex matrix, this highly simplified and protein-enzyme-free detection clearly demonstrates the huge potential of this generic HCR amplification system for sensitive and high-throughput multiplex target detection.

## 4. Conclusions

The Evalution™ platform was evaluated as a technology for highly sensitive and high-throughput multiplex nucleic acid analysis enabled by the unprecedented microparticle encoding capacity. To achieve the highest possible sensitivity, HCR was introduced as a universal signal amplification tool, benefiting from an isothermal and protein enzyme-free amplification. Singleplex detection of a nucleic acid target (HAB) indicated that different reaction times led to different levels of sensitivity, improving the limit of detection from 10^3^-fold after 10 min to 10^4^-fold after 60 min, compared to a non-amplified reference system. Varying the HCR reaction time offers the possibility to tune the assay for assay speed and/or sensitivity, as desired. The potential of sensitive and specific multiplex detection was further demonstrated for a multiplex panel of six different nucleic acid target molecules. This model system included both viruses (HAB, HAD and HB) and bacteria (SP, SPR1 and SPR2). The ability to distinguish between these two categories, as well as different virus strains (HA and HB), virus subtypes (HAB and HAD) and antibiotic susceptible bacterial strains (SP) from resistant strains (SPR1 and SPR2), is diagnostically relevant as specific species and their quantity are often associated with a unique disease management and treatment. A difference in assay performance was noticed for different target sequences. This difference can be attributed to the difference in the target and detection sequences, affecting hybridization efficiency and in some cases imposing undesired secondary structures.

In buffer conditions, detection limits as low as 33 ± 4, 51 ± 5; 106 ± 9, 88 ± 6, 151 ± 12 and 136 ± 11 fM were calculated based on the detection of HAB; HAD, HB, SP, SPR1 and SPR2 targets, respectively. Lastly, the direct detection of the HAB target in clinically relevant nasopharyngeal swab samples was demonstrated without the need for elaborate sample preparation steps. Using the highly simplified and protein-enzyme-free detection method, a calculated detection limit of 309 ± 80 fM was reached in a 10-fold diluted complex sample matrix, approximating the low fM levels detectable with the current gold standard analysis method, being PCR [18]. It should be noted that the use of branched HCR could potentially lead to even more efficient signal amplification resulting in even lower limits of detection [25]. If we compare the results obtained for the developed assay with other commonly used multiplex technologies described in literature, we see that the obtained detection limit of 1.1 pg for the detection of the HAB target in the sample matrix exceeds the detection limit of a recent study using the xMAP approach in combination with PCR amplification that reports a detection limit between 0.05 and 0.01 ng of DNA [26]. The microarray approach commercialized by Raybiotech reaches a detection limit between 0.005 and 0.05 pg for protein targets. In addition, a recent study using a similar microarray system in combination with PCR amplification to detect DNA targets reports a detection limit of 0.4 fg [27], indicating that the sensitivity or a reported approach strongly varies for different assays. Although more sensitive, a drawback to the reported microarray approach is the limited multiplex capacity of up to 40 different capture molecules, which is required to be able to link the spot position to one particular target on the area of each subarray [28]. If we look at the multiplexing capacity of the xMAP system, involving microspheres internally dyed with a fluorophore allows as many as 100 unique codes to detect 100 different targets simultaneously [29]. Since the Evalution does not rely on spectral encoding but rather on a 10-digit physical code, the encoding capacity goes up to 1024 different codes. Additionally, compared to spectral encoding strategies, the digital code intrinsic to the Evalution system does not suffer from spectral overlap and cannot be altered by light-induced damage or chemical degradation. Moreover, the reported detection strategy can be applied for a wide range of target molecules in a multiplex format, being not only nucleic acid targets but also proteins, by linking the HCR initiator sequence to target-specific bioreceptor molecules such as antibodies or aptamers [14]. As such, this approach has the potential to improve early stage disease diagnosis through the combined detection of both protein and nucleic-acid-based biomarkers in a high-throughput fashion.

## Data Availability

Not applicable.

## Supplementary Materials

The following supporting information can be downloaded at: https://www.mdpi.com/article/10.3390/bios13010100/s1, Table S1: Oligonucleotide sequences used in this work; Table S2: Data HCR reaction over time for different HAB target concentrations; Figure S1: Kinetic profile of the HCR reaction; Figure S2: The calibration curve of the reference system; Figure S3: The contribution of the HCR components to the non-specific interaction; Figure S4: Color maps indicating levels of the signal-to-noise ratio. References [16,18,22,30] are cited in the Supplementary Materials.
