# Supplementary material for: Highly Sensitive Multiplex Detection of Molecular Biomarkers Using Hybridization Chain Reaction in an Encoded Particle Microfluidic Platform
Source: Biosensors (Basel). 2023 Jan 6;13(1):100. doi: 10.3390/bios13010100 (PMC9856145; doi:10.3390/bios13010100)
Supplement: Supplementary file 1 [file biosensors-13-00100-s001.zip › biosensors-2085255-supplementary.pdf]

Oligonucleotide sequences

The sequences used throughout the experiments can be found in Table S1. The target sequences of human adenovirus, human bocavirus and streptococcus were derived from information available in the literature[1,2] and the open access nucleotide sequence database, GenBank. The hybridization chain reaction system was inspired by the system described by Ge *et al.* [3]. The sequences of the hairpin ensemble were kept the same, while the initiator sequences were redesigned with a recognition part for each of the target sequences.

Table S1. Oligonucleotide sequences used in this work.

**Name**	**Sequence (5′ → 3′)**
Human adenovirus type B (HAB)	TACCAGCAGCAC-CTCACCCTCTTCCCAACTCTGGTAC
Human adenovirus type D (HAB)	CTCTCAGATGTCAAAGAGGCTCCGGGTGGAA-GATGAC
Human bocavirus (HB)	CAAGTCCTCCAAACTCACCTGCGAGCTCTG-TAAGTACTATTAC
Streptococcus Pneumonia (SP)	AGGTAGTAAGGGAAACAATATCAAACTGAC-CATTGATTTGGCTTTCCAA
Resistant *Streptococcus Pneumonia* (SPR1)	TTCAGCTCCAATTTATTCTTGGTATAAATTGG-CATATGGATCTTTTCCT
Resistant *Streptococcus Pneumonia* (SPR2)	TCAGACCTATCAACCCAATATGTTT-GTTTTAACTAACAATTTAGAATCC
HAB detection probe	NH2-GTACCAGAGTTGGGAAGA
HAD detection probe	NH2-GTCATCTTCCACCCGGAG
HB detection probe	NH2-GTAATAGTACTTACAGAGCTC
SP detection probe	NH2-TTGGAAAGCCAAATCAATGGTCAG
SPR1 detection probe	NH2-AGGAAAAGATCCATATGCCAATTT
SPR2 detection probe	NH2-GGATTCTAAATTGTTAGTTAAAAC
HAB initiator	GGTGAGGTGCTGCTGGTAAATT-GGGTGCGGCTTAGGATCTGA
HAD initiator	CTCTTTGACATCTGAGAGAATT-GGGTGCGGCTTAGGATCTGA
HB initiator	CAGGTGAGTTTGGAGGACTTGAATT-GGGTGCGGCTTAGGATCTGA
SP initiator	TTGATATTGTTTCCCTTACTACCTAATT-GGGTGCGGCTTAGGATCTGA
SPR1 initiator	TACCAAGAATAAATTGGAGCTGAAAATT-GGGTGCGGCTTAGGATCTGA
SPR2 initiator	AACATATTGGGTTGATAGGTCTGAAATT-GGGTGCGGCTTAGGATCTGA
H1	GTGCGGCTTAG-GATCTGATGAAACTCAGATCCTAAGCCG-CACCCAATT
H2	GTTTCATCAGATCCTAAGCCGCACAATT-GGGTGCGGCTTAGGATCTGA-ATTO550

Hybridization chain reaction (HCR) durations

To evaluate the effect of different signal amplification times on the calculated LOD values, the HCR reaction was monitored over time for different HAB target concentrations (250, 50, 10, 2, 0.4 pM and a blank) (Figure S1). The reaction was monitored for a duration of 60 min with 5 min intervals. The channel background signal caused by the presence of the fluorescent hairpin component (H2) was subtracted for each of the concentrations. The background subtracted values for 10, 20, 30, 45 and 60 min were calculated and used to establish the corresponding calibration curves and the LOD (Figure 3).

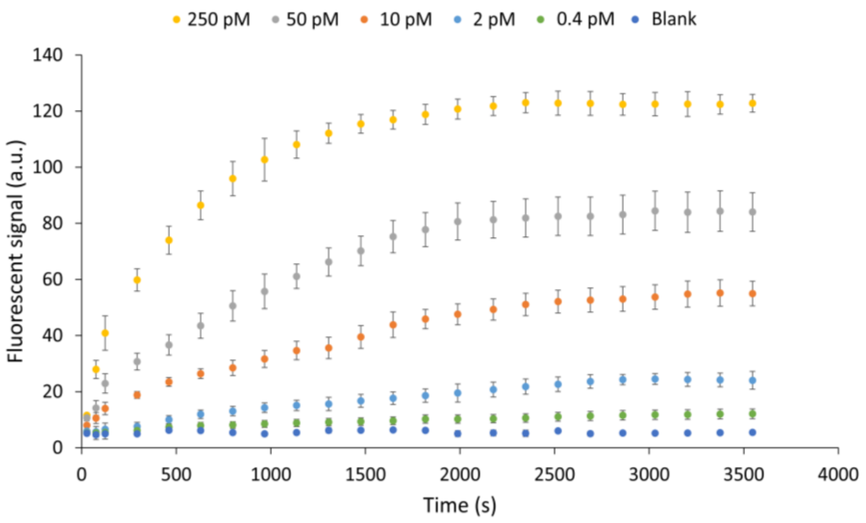

Figure S1. HCR reaction over time for different HAB target concentrations (250, 50, 10, 2, 0.4 pM and a blank). The error bars represent the standard deviation of three repetitions.

Table S2. Data HCR reaction over time for different HAB target concentrations .

Time	250 pM	50 pM	10 pM	2 pM	0.4 pM	Blank
60 min	122.8	84.4	54.9	24.1	12.1	5.5
50 min	122.5	84.5	53.7	24.5	11.8	5.2
40 min	122.9	82.5	52.1	22.6	11.1	6.0
30 min	118.8	77.7	45.9	18.6	10.2	6.1
20 min	112.2	66.2	35.6	15,6	9.1	6.0
10 min	86.4	43.5	26.4	11.9	7.9	6.2

Reference system calibration curve

To truly assess the added value of the amplification strategy on the signal, the results were compared to those of a non-amplified reference system. Different concentrations of HAB target were detected, ranging from 250 to 2 nM and a blank. This reference was established by flowing each of the two hairpin sequences sequentially instead of flowing them at same the time. This way, full control of the number of HCR cycles was achieved. In this case, as we wanted to limit the number of cycles to only one (k = 1), both hairpin sequences were flowed individually and separated in a washing step. In this reference system, only a single fluorescent label is present per target as after one cycle of HCR, only one fluorescent label was incorporated. By fitting the resulting calibration curve, a calculated LOD of 890 ± 110 pM was obtained (Figure S2).

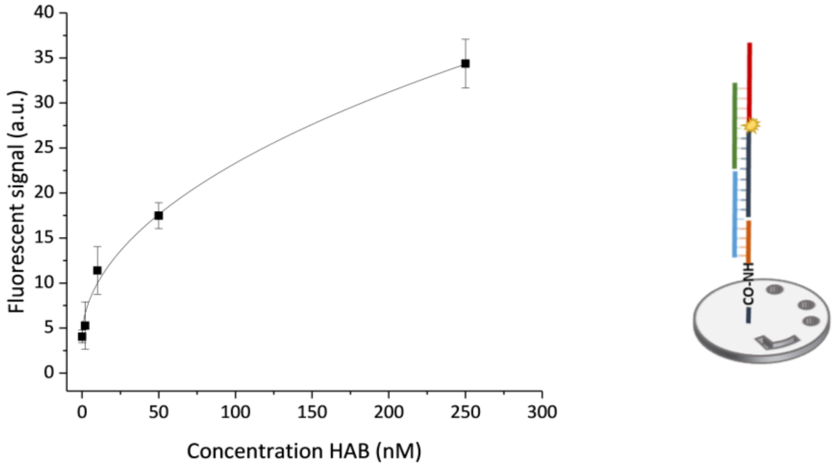

Figure S2. Calibration curve for a non-amplified reference system (singleplex). Different concentrations of HAB target were detected, ranging from 250 to 2 nM and a blank. The error bars represent the standard deviation of three repetitions.

Non-specific interactions in multiplex detection

After successful singleplex detection, the performance of the HCR system was evaluated in a multiplex environment. Preliminary experiments at a temperature of 25°C revealed that only a limited amount of non-specific interactions occurred in the absence of target and/or initiator (Figure S3). These interactions were neglected as they varied within an acceptable range from the fluorescent values attributed to the intrinsic microparticle autofluorescence. These results indicate that the majority of non-specific signal was caused by the non-specific interaction between the targets and detection probes, which is why we focused on optimizing this step. Varying the temperature in other assay steps might even further lower the number of non-specific interactions.

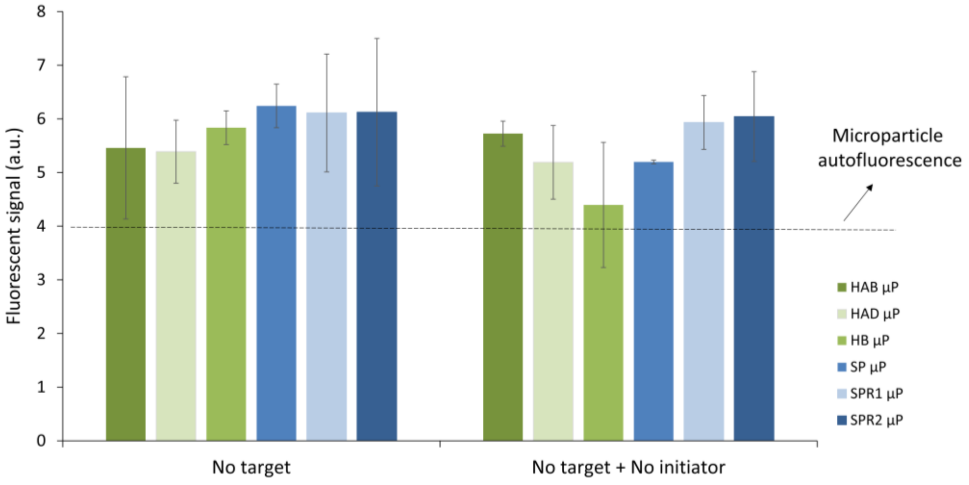

Figure S3. Non-specific interactions in the absence of target at 25°C. Each channel contained six different microparticle (µP) populations (HAB-, HAD-, HB-, SP-, SPR1- and SPR2- µP), and the different components of the HCR reaction were flowed, except for the target and the target and initiator. The non-specific interactions were determined by monitoring the fluorescent signal. The error bars represent one standard deviation (n=3)n.

To overcome the non-specific interactions occurring between the target and detection probes, elevated temperatures were tested during the target capture step, ranging from 35°C to 55°C, with a 5°C interval. The included temperature control unit allowed the Evalution™ device to apply specific temperatures for different assay steps. A concentration of 250 pM was administered for six different targets, including HAB, HAD, HB, SP,

SPR1 and SPR2, and each of them were in separate channels. All of the channels contained six different microparticle (µP) populations functionalized using different detection probes. The signal-to-noise ratios were obtained by dividing the specific signal by the non-specific signal of the five other microparticle populations and expressing it as a percentage, as visualized in Figure S4. The maximal signal-to-noise ratio was found for a temperature of 45°C.

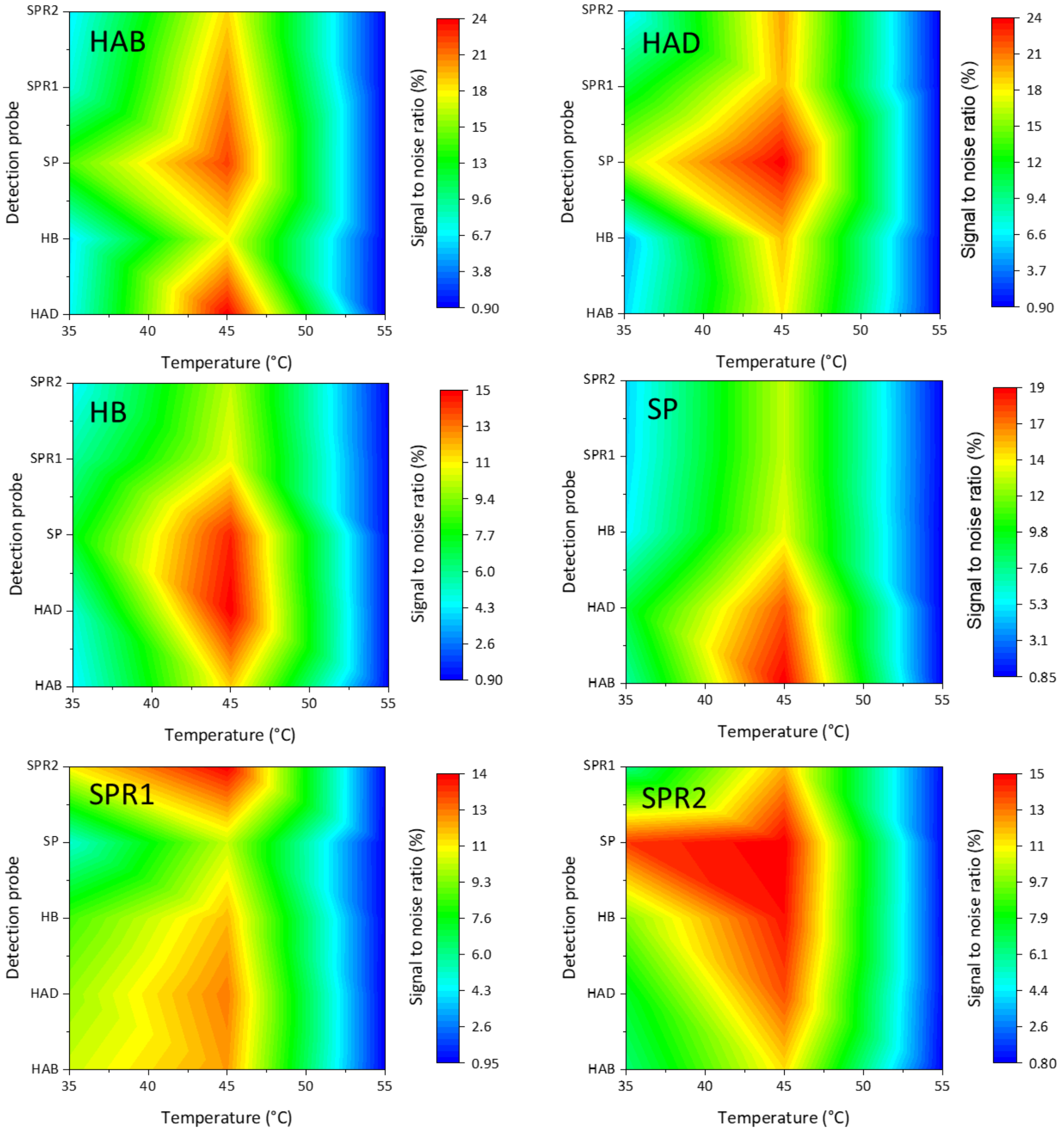

Figure S4. Color maps visualizing the signal-to-noise ratio for different temperatures of the target capture step, ranging between 35 and 55°C, with a 5°C interval. One target sequence, either HAB, HAD, HB, SP, SPR1 or SPR2, with a concentration of 250 pM was incubated per channel. Each channel contained six different microparticle populations, which were all functionalized using a different detection probe (specific for their corresponding target). For each target-specific µP population, the five non-specific probes are represented on the y-axis. The corresponding signal-to-noise ratio of the target-specific populations compared to each non-specific probe is shown as a color map for different incubation temperatures tested (x-axis). The red color indicates the highest signal-to-noise-ratio, and dark blue color represents the lowest ratio.

LOD calculation

The method, as described by Holstein et al.[4], begins by us identifying the signal threshold at which there is a probability of generating a false positive result, which is typically identified as L_D (i.e. the limit of detection in the signal domain). To calculate L_D, the limit of the blank L_C needs to be determined according to the following formula[4]:

$$L_C = \mu_{\text{blank}} + t(1 - \alpha, n - 1)\sigma_{\text{blank}}$$

Where μ_{blank} is the mean signal intensity and σ_{blank} is the standard deviation of n blank replicates which are multiplied with the 1 – α percentile of the t-distribution with n-1 degrees of freedom. The significance level α was chosen to be 0.05. The limit of detection in the signal domain L_D can be calculated with following equation[4]:

$$L_D = L_C + t[1 - \beta, m(n - 1)]\sigma_{\text{test}}$$

Where σ_{test} is the pooled standard deviation of the test replicates (n), which is multiplied with the 1 – β percentile of the t-distribution with m(n-1) degrees of freedom. The significance level β was chosen to be 0.05.

Next, the 4PL calibration curve is used to translate the L_D value in the signal domain to an LOD estimate in the concentration domain. Once the specific 4PL fit has been determined, it can be used to calculate the LOD by rearranging the equation as a function of the signal intensity (S) and plugging the L_D value as an assay signal[4].

$$\log(\text{LOD} + 2) = \hat{c}\left[\left(\frac{\hat{a} - \hat{d}}{L_D - \hat{d}}\right) - 1\right]^{1/\hat{b}}$$

This returns the LOD estimate, which is on the log(C+2) scale. In order to obtain the final LOD estimate on the concentration scale, the result is raised to the power of 10 and subtracted by 2.

References

1. K. Pehler-Harrington, M. Khanna, C. R. Waters and K. J. Henrickson, 'Rapid detection and identification of human adenovirus species by Adenoplex, a multiplex PCR-enzyme hybridisation assay', *J. Clin. Microbiol.*, 2004, **42**, 4072–4076.
2. Y. Zhang, D. Isaacman, R. Wadowsky, J. Rydquist-White, J. Post and G. Ehrlich, 'Detection of Streptococcus pneumoniae in whole blood by PCR', *J. Clin. Microbiol.*, 1995, **33**, 596–601.
3. Z. Ge, M. Lin, P. Wang, H. Pei, J. Yan, J. Shi, Q. Huang, D. He, C. Fan and X. Zuo, 'Hybridisation chain reaction amplification of MircoRNA detection with a tetrahedral DNA nanostructure-based electrochemical biosensor', *Anal. Chem.*, 2014, **86**, 2124–2130.
4. Holstein, C. A.; Griffin, M.; Hong, J.; Sampson, P. D. Statistical Method for Determining and Comparing Limits of Detection of Bioassays. Anal. Chem. 2015, **87**, 9795–9801.
